# Real-world daptomycin use across wide geographical regions: results from a pooled analysis of CORE and EU-CORE

**DOI:** 10.1186/s12941-016-0130-8

**Published:** 2016-03-15

**Authors:** R. Andrew Seaton, Armando Gonzalez-Ruiz, Kerry O. Cleveland, Kimberly A. Couch, Rashidkhan Pathan, Kamal Hamed

**Affiliations:** Queen Elizabeth University Hospital, Glasgow, UK; Darent Valley Hospital, Dartford, UK; University of Tennessee Health Science Center, Memphis, TN USA; Infectious Diseases Pharmacy Associates, Inc., Stevensville, MD USA; Novartis Healthcare Pvt. Ltd., Hyderabad, India; Novartis Pharmaceuticals Corporation, East Hanover, NJ USA

**Keywords:** CORE, Daptomycin, Efficacy, EU-CORE, Gram-positive infections, High dose, MRSA, Real-world, Safety, *Staphylococcus aureus*

## Abstract

**Background:**

Pooled data from two large registries, Cubicin^®^ Outcomes Registry and Experience (CORE; USA) and European Cubicin^®^ Outcomes Registry and Experience (EU-CORE; Europe, Latin America, and Asia), were analyzed to determine the characteristics and clinical outcomes of daptomycin therapy in patients with Gram-positive infections across wide geographical regions.

**Methods:**

Patients receiving at least one dose of daptomycin between 2004 and 2012 for the treatment of Gram-positive infections were included. Clinical success was defined as an outcome of ‘cured’ or ‘improved’. Post-treatment follow-up data were collected for a subset of patients (CORE: osteomyelitis and orthopedic foreign body device infection; EU-CORE: endocarditis, intracardiac/intravascular device infection, osteomyelitis, and orthopedic device infection). Safety was assessed for up to 30 days after daptomycin treatment.

**Results:**

In 11,557 patients (CORE, 5482; EU-CORE, 6075) treated with daptomycin (median age, 62 [range, 1–103] years), the most frequent underlying conditions were cardiovascular disease (54.7 %) and diabetes mellitus (28.0 %). The most commonly treated primary infections were complicated skin and soft tissue infection (cSSTI; 31.2 %) and bacteremia (21.8 %). The overall clinical success rate was 77.2 % (uncomplicated SSTI, 88.3 %; cSSTI, 81.0 %; osteomyelitis, 77.7 %; foreign body/prosthetic infection (FBPI), 75.9 %; endocarditis, 75.4 %; and bacteremia, 69.5 %). The clinical success rate was 79.1 % in patients with *Staphylococcus aureus* infections (MRSA, 78.1 %). An increasing trend of high-dose daptomycin (>6 mg/kg/day) prescribing pattern was observed over time. Clinical success rates were higher with high-dose daptomycin treatment for endocarditis and FBPI. Adverse events (AEs) and serious AEs possibly related to daptomycin therapy were reported in 628 (5.4 %) and 133 (1.2 %) patients, respectively.

**Conclusions:**

The real-world data showed that daptomycin was effective and safe in the treatment of various Gram-positive infections, including those caused by resistant pathogens, across wide geographical regions.

## Background

Resistant Gram-positive pathogens such as methicillin-resistant *Staphylococcus aureus* (MRSA) and vancomycin-resistant enterococci (VRE) are associated with increased morbidity, mortality, and healthcare expenditures in hospitalized patients [1, 2]. Vancomycin is known to be an active agent for the treatment of Gram-positive infections, but there are concerns regarding its declining efficacy, potentially due to the “minimum inhibitory concentration creep” in MRSA [1, 3]. Therapeutic options such as clindamycin, co-trimoxazole, fluoroquinolones, minocycline, or the addition of rifampin may be useful, however, their use is limited to patients without life-threatening infections [4, 5]. Moreover, resistance to linezolid has been reported and its prolonged exposure can lead to myelosuppression [6, 7]. Other antibiotics with MRSA activity such as ceftaroline, ceftobiprole, telavancin, and tigecycline represent alternatives for the treatment of infections caused by drug-resistant Gram-positive pathogens [8]. However, there are some reports of safety issues associated with these antibiotics; hematological disorders and rash with ceftaroline, gastrointestinal upset with ceftobiprole, nephrotoxicity with telavancin, and pancreatitis with tigecycline [9–12].

Treatment choices are increasing for Gram-positive infections, including those caused by resistant pathogens. Various agents have either been recently approved (e.g. tedizolid, oritavancin) [8] or are under development, reflecting the need for “niche” antibiotics, particularly for difficult-to-treat infections [4, 13, 14].

Daptomycin is a cyclic lipopeptide with rapid bactericidal activity against a wide range of Gram-positive pathogens such as methicillin-susceptible *S. aureus* (MSSA), MRSA, and VRE [15, 16]. Daptomycin is approved in adult patients for the treatment of complicated skin and soft tissue infection (cSSTI; 4 mg/kg/day), right-sided infective endocarditis (RIE) due to *S.* *aureus*, and bacteremia associated with cSSTI or RIE (6 mg/kg/day) [17, 18]. However, high-dose (>6 mg/kg/day) daptomycin is often recommended for difficult-to-treat infections [14, 19–22]. Several study reports showed that high-dose daptomycin is increasingly used in patients with various deep seated infections and in those failing treatment with other antibiotics [23–26]. In addition to the approved indications, daptomycin has shown to be effective in the treatment of other infections, such as left-sided infective endocarditis (LIE), osteomyelitis, and orthopedic device infections [27–29]. Randomized controlled trials have shown a favorable safety and efficacy profile of daptomycin in patients with *S. aureus* bacteremia, endocarditis, and osteomyelitis/orthopedic device infections [30–32].

The Cubicin^®^ Outcomes Registry and Experience (CORE) and the European Cubicin^®^ Outcomes Registry and Experience (EU-CORE), both multicenter, retrospective, non-interventional registries, were conducted to collect real-world data on the characteristics and clinical outcomes of patients receiving daptomycin [33, 34]. CORE included data from approximately 164 sites in the United States of America (USA) [35–37], whereas EU-CORE comprised data from 310 sites across 18 countries in Europe (12), Latin America (5), and Asia (1). Various independent reports on the results from CORE (2004–2009) and EU-CORE (2006–2012) have been published [33–36, 38]. The results of CORE and EU-CORE showed that daptomycin is also used for treating infections other than those approved [35, 39]. Combining data from two real-world registries conducted in different regions may help in understanding the trend of prescribing patterns and duration of treatment versus effectiveness and safety in a large number of patients treated with daptomycin between 2004 and 2012.

## Methods

### Patients and data collection

The protocols were approved by the health authority and the Institutional Review Board (IRB) or Ethics Committee (EC) of each participating country. The methodologies have been published previously [36, 40]. Written informed consent was obtained from patients according to the requirements of the IRB or EC and/or the local data privacy regulations. Patients who had received at least one dose of daptomycin between January 2004 and April 2012 for treatment of Gram-positive bacterial infections were included. Patients should have been followed up for 30 days after treatment. Patients who had received daptomycin as part of a controlled clinical trial were not eligible. In CORE, patients with osteomyelitis or orthopedic foreign body device infection (enrolled between 2007 and 2008) were evaluated at the end of daptomycin treatment; those with at least one post-treatment follow-up assessment were also included in the follow-up data collection up to 2009. In EU-CORE, patients with endocarditis, intracardiac/intravascular device infection, osteomyelitis, or orthopedic device infection were followed for up to 2 years from 2012 to 2014. In both registries, the overlapping data collection period was from 2007 to 2009. Demographic, antibiotic, microbiologic, and clinical data were recorded retrospectively using standardized case report forms as per the protocols. This analysis extracted data from all patients who received daptomycin from 2004 to 2012 (CORE, 2004–2009; EU-CORE, 2006–2012).

### Definitions

Clinical outcomes were assessed by investigators at the end of daptomycin treatment based on the following protocol-defined criteria: cured, clinical signs and symptoms resolved, no additional antibiotic therapy was necessary, or infection cleared with a negative culture reported; improved, partial resolution of clinical signs and symptoms and/or additional antibiotic therapy was warranted; failed, inadequate response to daptomycin therapy, worsening or new/recurrent signs and symptoms, need for a change in antibiotic therapy, or a positive culture reported at the end of the therapy; and non-evaluable, unable to determine response due to insufficient information. Clinical success was defined as an outcome of ‘cured’ or ‘improved’. Time to improvement was recorded. The reasons for discontinuation of daptomycin therapy and details of other antibiotics prescribed concomitantly or following daptomycin were also recorded. Among all registry-enrolled patients, the safety population comprised patients for whom any safety parameters were assessed, and the efficacy population comprised patients for whom clinical outcomes were assessed. All adverse events (AEs) were reported, regardless of their relationship to daptomycin; the severity of these AEs was determined by the investigators.

### Statistical analysis

Statistical analysis was performed using SAS version 9.3 (SAS Institute Inc., Cary, NC, USA). Due to the nature of the two observational uncontrolled registry trials, inferential analyses were not conducted and no formal statistical methodology, except simple descriptive statistics, was used. All analyses were considered to be explanatory. Numerical variables were summarized as arithmetic mean, standard deviation, median, minimum, first quartile, third quartile, and maximum for the continuous variables, whereas the categorical variables were summarized according to absolute and relative frequencies.

Logistic regression analysis was performed to assess risk factors for CPK elevation, with CPK elevation (yes/no) as response variable and the following variables as covariates: age, use of statin therapy, history of renal disease or diabetes mellitus, significant underlying disease, infection type, initial dose level, surgical procedure, concomitant use of antibiotics, creatinine clearance (CrCl), and baseline CPK.

## Results

### Patient demographics and clinical characteristics

A total of 11,557 patients (CORE, 5482; EU-CORE, 6075) treated with daptomycin were included in this analysis. Baseline patient demographic and clinical characteristics by overall pooled data and individual studies during the overlapping period from 2007 to 2009, are summarized in Table 1. The median age of the patients was 62 (range, 1–103) years, with 38.4 % aged ≥65 years, and a majority (88.7 %) having significant underlying diseases. The most common underlying conditions were cardiovascular disease (54.7 %) and diabetes mellitus (28.0 %), followed by malignancy (16.7 %) and renal disease (16.6 %). Daptomycin was used to treat a wide range of primary infections (Table 2). The commonly treated primary infections were cSSTI (31.2 %), bacteremia (21.8 %), foreign body/prosthetic infection (FBPI; 8.5 %), osteomyelitis (8.6 %), and endocarditis (6.9 %; including 3.9 % with LIE). The most common secondary infections were bacteremia (3.8 %), cSSTI (2.6 %), osteomyelitis (1.7 %), and endocarditis (1.2 %). Overall, 14.5 % of patients had renal impairment (CrCl <30 mL/min) and 1095 (9.5 %) patients were on dialysis at the time of initiation of daptomycin therapy.

**Table 1:** Baseline patient demographic and clinical characteristics

Characteristic	Pooled data (2004–2012) N = 11,557 n (%)	Overlapping time period (2007–2009)	
		CORE^a^	EU-CORE^a^
		N = 2827 n (%)	N = 3333 n (%)
Male	6587 (57.0)	1454 (51.4)	2150 (64.5)
Age^b^ (years), median (range)	62.0 (1–103)	56.5 (4–99)	63.0 (1–103)
<65 years	7111 (61.5)	1945 (68.8)	1758 (52.7)
≥65 years	4441 (38.4)	882 (31.2)	1572 (47.2)
≥75 years	1732 (15.0)	265 (9.4)	734 (22.0)
Body weight (kg), median (range)	78.0 (6–275)	81.9 (15–259)	75.0 (6–200)
Race, Caucasian	7191 (62.2)	1967 (69.6)	3058 (91.7)
Setting prior to daptomycin therapy^c^			
Hospital	7278 (63.0)	1359 (49.3)	2558 (76.7)
Nursing home/extended care	431 (3.7)	152 (5.4)	102 (3.1)
Community	3772 (32.6)	1275 (45.1)	637 (19.1)
Other	57 (0.5)	5 (0.2)	24 (0.7)

Data are presented as n (%), unless indicated otherwise

^a^Data are from three overlapping periods: 2007–2009

^b^Data missing for 5 patients

^c^Data missing for 19 patients

**Table 2:** Primary infections and pathogens

Primary infection	Pooled data (2004–2012) N = 11,557 n (%)	Overlapping time period (2007–2009)	
		CORE^a^	EU-CORE^a^
		N = 2827 n (%)	N = 3333 n (%)
Complicated skin and soft tissue infection	3607 (31.2)	803 (28.4)	1092 (32.8)
Bacteremia	2522 (21.8)	642 (22.7)	741 (22.2)
Endocarditis	798 (6.9)	103 (3.6)	338 (10.1)
Foreign body/prosthetic infection	988 (8.5)	217 (7.7)	294 (8.8)
Osteomyelitis (non-prosthetic and prosthetic device-related)	994 (8.6)	281 (9.9)	193 (5.8)
Uncomplicated skin and soft tissue infection	1510 (13.1)	475 (16.8)	334 (10.0)
Other^b^	1138 (9.8)	306 (10.8)	341 (10.2)

Primary pathogen	Pooled data (2004–2012) N = 7912 n (%)	Overlapping time period (2007–2009)	
		CORE^a^	EU-CORE^a^
Positive culture^c^		N = 2002 n (%)	N = 2240 n (%)
*Staphylococcus aureus*	3673 (46.4)	925 (46.2)	931 (41.6)
Methicillin-susceptible	1104 (14.0)	208 (10.4)	356 (15.9)
Methicillin-resistant	2261 (28.6)	621 (31.0)	509 (22.7)
Susceptibility unknown	308 (3.9)	96 (4.8)	66 (2.9)
CoNS	1690 (21.4)	276 (13.8)	677 (30.2)
*Staphylococcus epidermidis*	850 (10.7)	103 (5.1)	390 (17.4)
*Staphylococcus* spp.–coagulase negative	840 (10.6)	173 (8.6)	287 (12.8)
Enterococci	1380 (17.4)	495 (24.7)	255 (11.4)
Vancomycin-resistant	576 (7.2)	284 (14.2)	36 (1.0)
*Enterococcus faecalis*	492 (6.2)	134 (6.7)	125 (5.6)
Vancomycin-resistant	83 (1.0)	37 (0.5)	10 (0.3)
*Enterococcus faecium*	684 (8.6)	277 (13.8)	110 (4.9)
Vancomycin-resistant	493 (6.2)	247 (12.3)	26 (0.8)
*Enterococcus spp.*	204 (2.6)	84 (4.2)	20 (0.9)
Streptococci	326 (4.1)	71 (3.5)	98 (4.4)
Other^d^	843 (10.7)	235 (11.7)	279 (12.5)

*CoNS* coagulase-negative staphylococci

^a^Data are from three overlapping periods: 2007–2009

^b^Includes septic arthritis, urinary tract infections/pyelonephritis, central nervous system infections, metastatic abscess, antibiotic prophylaxis (surgical and non-surgical), neutropenic fever, necrotizing fasciitis, necrotizing infections, unknown or not otherwise specified infections, and data missing

^c^Percentage is calculated based on positive culture results

^d^Includes *Clostridium difficile*, *Clostridium perfringens*, *Clostridium* species, *Corynebacterium* species, *Staphylococcus* species coagulase not specified, Gram-positive and Gram-negative bacilli and cocci, *Leuconostoc* species, *Peptococcus* species, *Peptostreptococcus species,* fungi/yeast, viruses, and organisms with invalid/ambiguous pathogen code

### Microbiology

Culture results were available for a total of 9664 (83.6 %) patients, of whom 7912 (81.9 %) had positive culture results and 1752 (18.1 %) had negative culture results (Table 2). The most frequently isolated pathogens in patients with positive culture results were *S. aureus* (46.4 %, including 28.6 % MRSA and 14.0 % MSSA), coagulase-negative staphylococci (CoNS; 21.4 %), and enterococci (17.4 %; VRE, 7.3 %).

### Previous and concomitant therapies

The majority of patients (n = 8221; 71.1 %) received prior antibiotic therapy, most commonly glycopeptides (34.3 %; vancomycin, 30.6 %; teicoplanin, 4.2 %), penicillins (19.5 %), cephalosporins (15.6 %), and fluoroquinolones (13.9 %). The most common reasons for switching to daptomycin included failure of prior antibiotic therapy (28.0 %), resistant or non-susceptible Gram-positive organism (10.7 %), and narrow antibiotic spectrum (10.0 %). Daptomycin was used concomitantly with other antibiotics in 6631 (57.4 %) patients who received treatment in an inpatient setting; the frequently administered concomitant antibiotics were carbapenems (17.6 %), penicillins (12.1 %), fluoroquinolones (11.5 %), and cephalosporins (10.0 %).

### Daptomycin prescribing patterns

**Fig. 1:**
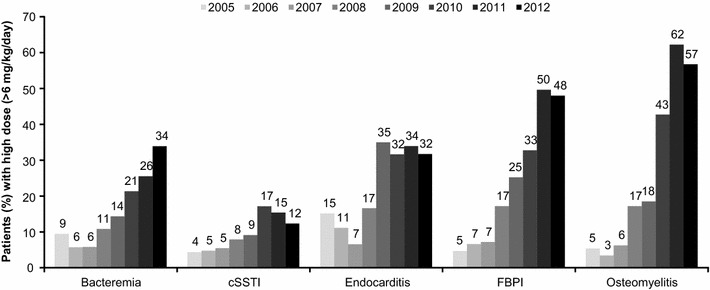
Prescribing pattern of high-dose daptomycin over time. *cSSTI* complicated skin and soft tissue infection, *FBPI* foreign body/prosthetic infection

The most commonly prescribed dose of daptomycin was 6 mg/kg/day (n = 4968; 43.0 %), followed by 4 mg/kg/day (n = 3469; 30.0 %). A similar daptomycin prescribing pattern was noted in the overlapping period of CORE (6 mg/kg/day [48.5 %], followed by 4 mg/kg/day [26.6 %]) and EU-CORE (6 mg/kg/day [45.5 %], followed by 4 mg/kg/day [29.9 %]). In particular, 54.2 % of patients with bacteremia and 42.0 % patients with cSSTI were treated with daptomycin 6 mg/kg/day and 4 mg/kg/day, respectively. A total of 1564 (13.5 %) patients received high-dose daptomycin (>6 mg/kg/day). A trend toward use of higher doses (>6 mg/kg/day) over time was observed for most of the infections, particularly in patients with bacteremia, osteomyelitis, and FBPI (Fig. 1).

**Fig. 2:**
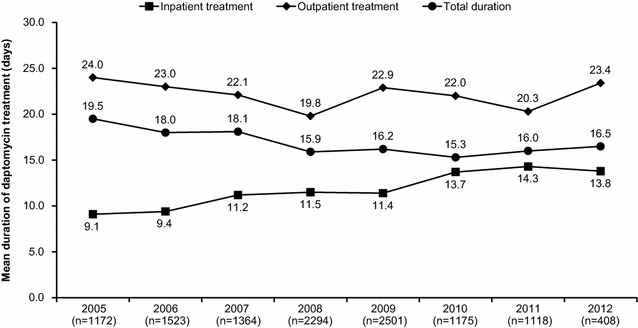
Mean durations of daptomycin treatment according to inpatient and outpatient treatment settings over time (2005–2012)

**Fig. 3:**
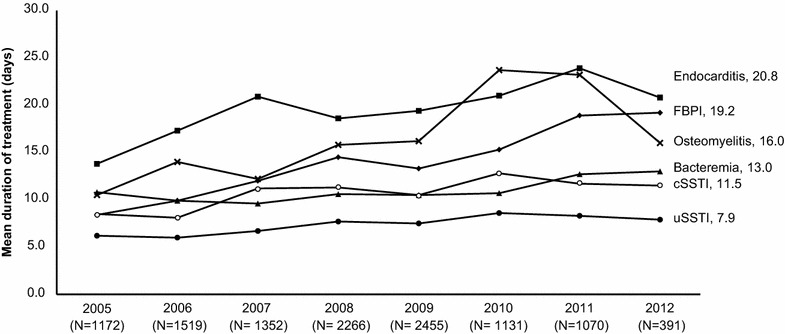
Mean durations of daptomycin treatment by primary infection over time (2005–2012). *cSSTI* complicated skin and soft tissue infection, *FBPI* foreign body/prosthetic infection

Overall, the median duration of treatment with daptomycin was 12.0 (range, 1–370) days; 8.0 (range, 1–246) days in the inpatient setting and 16.0 (range, 1–358) days in the outpatient setting. The mean duration of daptomycin treatment according to inpatient/outpatient treatment settings over time (years) is shown in Fig. 2. The duration of treatment was lower in inpatients compared with outpatients. Among the key primary infections, the median inpatient treatment duration was longest for endocarditis at 16.0 (range, 1–112) days and shortest for uncomplicated SSTI (uSSTI) at 6.0 (range, 1–56) days. The mean durations of daptomycin treatment by primary infection over time are shown in Fig. 3. The duration of treatment was higher for endocarditis, FBPI, and osteomyelitis as compared with uSSTI, cSSTI, and bacteremia. An increasing trend was observed in mean duration of treatment for osteomyelitis, FBPI, and endocarditis over time.

A total of 6471 (56.0 %) patients completed the daptomycin treatment; however, 23.4 % switched therapy (e.g., stepped-down to oral antibiotic therapy). Discontinuation of daptomycin therapy due to AEs and treatment failure was reported in 4.6 and 3.3 % of patients, respectively.

### Clinical outcomes

**Fig. 4:**
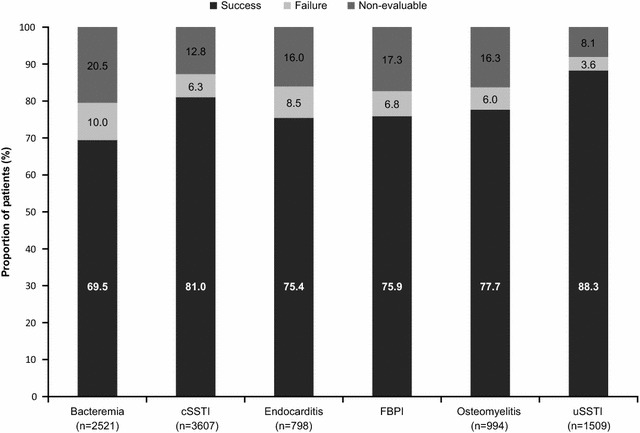
Clinical outcomes by primary infection. *cSSTI* complicated skin and soft tissue infection, *FBPI* foreign body/prosthetic infection, *uSSTI* uncomplicated skin and soft tissue infection

**Fig. 5:**
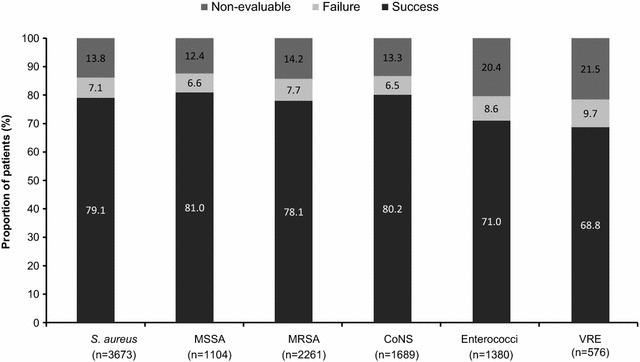
Clinical outcomes by primary pathogen. *CoNS* coagulase-negative staphylococci, *MRSA* methicillin-resistant *S. aureus*, *MSSA* methicillin-susceptible *S. aureus*, *VRE* vancomycin-resistant enterococci

An overall clinical success rate of 77.2 % was reported with daptomycin therapy. The clinical success rates ranged from 69.5 % for bacteremia to 88.3 % for uSSTI (Fig. 4). The clinical success rates during the overlapping period (2007–2009) were similar overall and for the different primary infections. The success rates in patients with resistant pathogens such as MRSA and VRE were 78.1 and 68.8 %, respectively (Fig. 5).

**Fig. 6:**
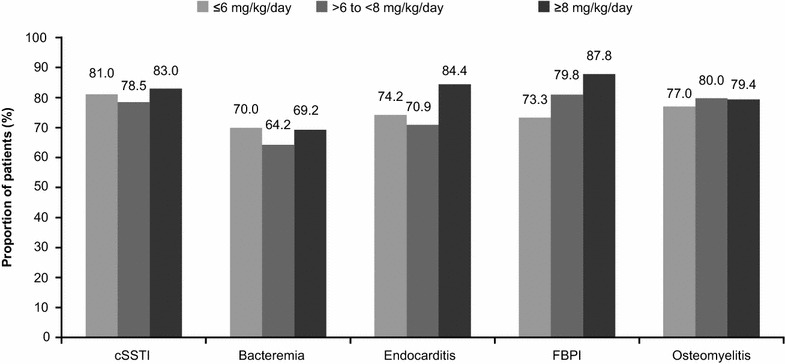
Clinical success rates for primary infection by daptomycin dose. *cSSTI* complicated skin and soft tissue infection, *FBPI* foreign body/prosthetic infection

The clinical success rate was slightly higher (80.3 %) when daptomycin was used as a first-line than that when used as a second-line therapy (75.9 %). For infections associated with MRSA as the primary pathogen, the clinical success rate with first-line daptomycin treatment (n = 841) was 83.7 % compared with 76.3 % with second-line daptomycin treatment (n = 2819). Clinical success rates tended to be higher with increasing daptomycin doses for endocarditis and FBPI (Fig. 6).

The patients who received daptomycin as monotherapy reported higher (82.7 %) clinical success rates than those who received concomitant antibiotic therapy (74.3 %).

### Safety

Daptomycin was generally well tolerated. AEs, regardless of their relationship to daptomycin treatment, were reported in 1879 (16.3 %) patients, and serious AEs (SAEs) were reported in 1050 (9.1 %) patients. Increased blood creatine phosphokinase (CPK; 1.9 %), multi-organ failure (1.0 %), and sepsis (1.0 %) were the most commonly reported AEs regardless of relationship to daptomycin treatment, and the most common SAEs were multi-organ failure (1.0 %), sepsis (1.0 %), and septic shock (0.7 %). AEs and SAEs possibly related to daptomycin treatment were reported in 628 (5.4 %) and 133 (1.2 %) patients, respectively. Elevated blood CPK levels in 175 (1.5 %) patients, myalgia in 21 (0.2 %) patients, rhabdomyolysis in 12 (0.1 %) patients, and both myopathy and eosinophilic pneumonia in 4 (0.03 %) patients each were reported as AEs possibly related to daptomycin treatment.

Blood CPK levels at baseline were measured in 4206 (36.4 %) patients. Of these, the majority (n = 3503; 83.3 %) had CPK levels ≤1 × upper limit of normal (ULN), 72 (1.7 %) had levels >5–10 × ULN, and 115 (2.7 %) had levels >10 × ULN. Blood CPK levels during daptomycin therapy were measured in 5024 (43.5 %) patients; of those, 3794 (75.5 %) had CPK levels ≤1 × ULN, 126 (2.5 %) had levels >5–10 × ULN, and 166 (3.3 %) had levels >10 × ULN.

**Fig. 7:**
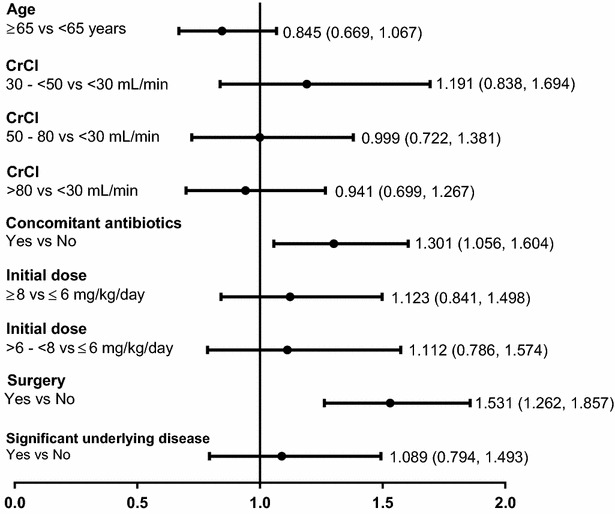
Logistic regression analysis of CPK elevation versus risk factors. Data are presented as odds ratio and 95 % confidence interval. *CrCl* creatinine clearance

Results of the logistic regression analysis to assess risk factors for CPK elevation showed that factors such as age, initial daptomycin dose and CrCl were not statistically significant at 5 % level of significance; however, surgical intervention and concomitant antibiotic therapy were found to be statistically significant at 5 % level of significance (Fig. 7). A total of 57 (1.7 %) patients experienced a shift of CPK elevation from ≤10 × ULN at baseline to >10 × ULN. AEs leading to study drug discontinuation occurred in 519 (4.5 %) patients, most frequently due to infections and infestations (1.0 %) including sepsis (0.3 %) and septic shock (0.3 %). A total of 674 (5.8 %) patients died during the daptomycin treatment; the most frequent causes of death were sepsis (0.9 %) and septic shock (0.7 %).

## Discussion

The CORE and EU-CORE pooled data analysis reflects the clinical experience with daptomycin use in a real-world setting. The results suggest that daptomycin is widely used to treat various infections caused by Gram-positive bacteria, including resistant strains (MRSA, CoNS, and VRE), in a complex patient population with multiple co-morbidities across the USA, Europe, Latin America, and Asia. Daptomycin demonstrated good safety and effectiveness outcomes, when used as second- or first-line therapy. Daptomycin was used to treat the approved indications (cSSTI, bacteremia, and RIE). In addition, patients with LIE, osteomyelitis, prosthetic joint infections, neutropenic fever, sepsis of unknown origin, and surgical site infections caused by Gram-positive pathogens were also treated, which reflects an unmet medical need of approved treatment options for these conditions. Several reports have suggested that daptomycin is effective and has an overall good safety profile in various clinical conditions such as SSTI, bacteremia, osteomyelitis and endocarditis in different geographical regions [15, 29, 41–43]. Rege et al. have reported good tolerability and high clinical success rates with daptomycin when administered for >14 days, in a patient population from the USA [34]. Similarly, the results from an 8-year clinical experience with daptomycin showed favorable safety and effectiveness profiles with lower overall clinical failure rates in Europe, Latin America, and Asia [33, 40, 43]. These results complement the previously published data from randomized clinical trials [31, 32]. Treatment with daptomycin in the real-world setting showed high clinical success rates across a range of pathogens, both for labeled (cSSTI, bacteremia, and RIE), and non-labeled (osteomyelitis, FBPI, and LIE) infections. High success rates with daptomycin treatment were observed irrespective of first-line or second-line use. In patients with MRSA infections, the clinical success rates were numerically higher with first-line (83.7 %) compared with second-line (76.3 %) daptomycin treatment. Increased use of first-line daptomycin treatment for suspected and confirmed resistant pathogens, such as MRSA, reflects increasing awareness of daptomycin use for resistant pathogens and the limitations of available treatment options [31, 32, 44]. This pooled analysis reinforces the data from the previously published real-world reports (CORE and EU-CORE) [33, 35].

Considering the linear pharmacokinetics and dose-dependent bactericidal activity of daptomycin [45], high dose (>6 mg/kg/day) is sometimes recommended to minimize the risk of resistance development in patients with difficult-to-treat infections including those caused by resistant pathogens (MRSA and VRE) [45–47]. Daptomycin has a long half-life of 8 h, and demonstrates a prolonged post-antibiotic effect of up to 6.8 h. It is highly bound to serum proteins (90 %) and is distributed primarily in the extracellular fluid. It effectively penetrates bone and inflamed soft tissues and, therefore, is efficacious in the treatment of deep tissue infections [18, 48].

A number of national and international guidelines recommend high-dose daptomycin (>6 mg/kg/day) as a possible therapeutic alternative for difficult-to-treat infections [20, 21, 49, 50]. In combination with other antibiotics, daptomycin (10 mg/kg/day) is also recommended for persistent MRSA bacteremia and vancomycin treatment failures [19, 20, 49, 51].

A study including 70 patients with IE receiving high-dose daptomycin (≥8 mg/kg/day) has shown successful clinical outcomes in all patients without any reports of discontinuation due to toxicity or AEs [52]. In a larger study of 250 patients with complicated Gram-positive infections, high-dose daptomycin (8 mg/kg/day) was reported to be effective and have a favorable safety profile [52]. Furthermore, various studies demonstrated that high-dose daptomycin is effective and well tolerated in the treatment of difficult-to-treat infections such as LIE, mediastinitis after cardiac surgery, and osteomyelitis [7, 52]. The results from the current analysis showed a trend toward the use of higher doses (>6 mg/kg/day) over time with high success rates for endocarditis and FBPI, which is also supported by previously published literature [7, 49, 52].

It is well recognized that daptomycin treatment is associated with blood CPK elevation, however, no significant correlation was reported between daptomycin dose and blood CPK elevation [14, 53, 54]. In the present analysis, a small proportion of patients experienced elevation in blood CPK levels; however, this was not always associated with adverse musculoskeletal effects. There was no correlation observed between blood CPK elevation and factors such as age, initial daptomycin dose, or CrCl. Furthermore, no new or unexpected safety findings were observed in this analysis. These safety results are consistent with earlier published real-world reports from the USA and Europe [34, 41, 55].

The registries have inherent limitations, such as the retrospective nature of the data collection and non-comparative, non-blinded, and non-randomized design. However, the registries allow the inclusion of diverse infections and the use of concomitant antibiotics, including broad-spectrum antibiotics. Therefore, the results mimic real-world clinical experience with daptomycin and expand the evidence derived from clinical trials.

The present analysis re-affirms the real-world safety and effectiveness of daptomycin across wide geographical regions including the USA and Europe. The data suggest that daptomycin is effective and well tolerated for the management of difficult-to-treat infections caused by various Gram-positive pathogens, including those caused by resistant species (MSSA, MRSA, CoNS, and VRE). Moreover, a trend of increased use of high-dose daptomycin was noted over time, specifically in patients with endocarditis, osteomyelitis, and prosthetic joint infections.
